# A cluster randomised trial of an intervention to increase the implementation of physical activity practices in secondary schools: study protocol for scaling up the Physical Activity 4 Everyone (PA4E1) program

**DOI:** 10.1186/s12889-019-6965-0

**Published:** 2019-07-04

**Authors:** Rachel Sutherland, Elizabeth Campbell, Nicole Nathan, Luke Wolfenden, David R. Lubans, Philip J. Morgan, Karen Gillham, Chris Oldmeadow, Andrew Searles, Penny Reeves, Mandy Williams, Nicole Evans, Andrew Bailey, Ross Morrison, Matthew McLaughlin, John Wiggers

**Affiliations:** 1 Hunter New England Population Health, Locked Bag 10, Wallsend, NSW 2287 Australia; 2 School of Medicine and Public Health, University of Newcastle, Newcastle, 2308 Australia; 3 Hunter Medical Research Institute, Newcastle, NSW 2300 Australia; 4 Priority Research Centre in Physical Activity and Nutrition, School of Education, University of Newcastle, Newcastle, NSW Australia; 5 South Western Sydney Local Health District, Locked Mail Bag 7279, Liverpool BC, NSW 1871 Australia; 6 Central Coast Local Health District, 4-6 Watt Street, Gosford, NSW 2250 Australia; 7 Mid North Coast Local Health District, P.O. Box 126, Port Macquarie, NSW Australia; 8 New South Waled Department of Education, Schools Sports Unit, Level 3, 1 Oxford Street, Darlinghurst, NSW 2010 Australia

**Keywords:** Physical activity, Adolescents, School, Randomised controlled trial, implementation, multi-component, scale-up

## Abstract

**Background:**

The implementation of interventions at-scale is required to maximise population health benefits. ‘Physical Activity 4 Everyone (PA4E1)’ was a multi-component school-based program targeting adolescents attending secondary schools in low socio-economic areas. An efficacy trial of the intervention demonstrated an increase in students’ mean minutes of moderate-to-vigorous physical activity (MVPA) per day and lower weight gain at low incremental cost. This study aims to assess the effectiveness and cost effectiveness of a multi-component implementation support intervention to improve implementation, at-scale, of the evidence based school physical activity (PA) practices of the PA4E1 program. Impact on student PA levels and adiposity will also be assessed, in addition to the cost of implementation.

**Methods:**

A cluster randomised controlled trial, utilising an effectiveness-implementation hybrid design, will be conducted in up to 76 secondary schools located in lower socio-economic areas across four health districts in New South Wales (NSW), Australia. Schools will be randomly allocated to a usual practice control arm or a multi-component implementation support intervention to embed the seven school PA practices of the PA4E1 program. The implementation support intervention incorporates seven strategies including executive support, in-School Champion, teacher training, resources, prompts, audit and feedback and access to an external Support Officer. The primary trial outcome will be the proportion of schools meeting at least four of the seven physical activity practices of the program, assessed via surveys with Head Physical Education teachers at 12 and 24-months. Secondary outcomes will be assessed via a nested evaluation of student PA and adiposity at 12-months (Grade 8 students) and 24 months (Grade 9 students) undertaken in 30 schools (15 per group). Resource use associated with the implementation intervention will be measured prospectively. Linear mixed effects regression models will assess program effects on the primary outcome at each follow-up period.

**Discussion:**

This study is one of few evidence-based multi-component PA programs scaled-up to a large number of secondary schools and evaluated via randomised controlled trial. The use of implementation science theoretical frameworks to implement the evidence-based program and the rigorous evaluation design are strengths of the study.

**Trial registration:**

Australian New Zealand Clinical Trials Registry ACTRN12617000681358 registered 12th May 2017. Protocol Version 1.

**Electronic supplementary material:**

The online version of this article (10.1186/s12889-019-6965-0) contains supplementary material, which is available to authorized users.

## Background

Physical inactivity is the fourth leading cause of mortality globally [1] and accrues a substantial negative economic impact [2]. Physical inactivity is a major risk factor in the development of non-communicable diseases, including coronary heart disease, type II diabetes and certain cancers [3]. Adolescence is considered a critical period, when physical inactivity adversely impacts on physical, mental and social health [4–6], and physical activity (PA) habits track into adulthood [7]. Despite the widely accepted benefits of PA during adolescence, less than 10% of adolescents globally (including Australia) accumulate sufficient PA to accrue the associated health benefits [8].

Secondary schools are a key setting to provide opportunities for PA, as they provide access to adolescents and their families for ongoing periods in a critical development phase. In addition, schools have the resources, professional skills of teachers and policies to encourage physically active lifestyles [9, 10]. School-based PA programs for children can be effective at increasing mean duration of daily moderate-to-vigorous physical activity (MVPA), the proportion of students meeting PA guidelines, and can reduce weight, as demonstrated in a number of systematic reviews [9, 11–13]. Additionally, whilst research is currently limited, there is also some evidence suggesting a sustained impact of the school-based PA programs beyond the initial intervention period [14]. However, few programs targeting adolescents have had beneficial effects on whole day PA [15–18], particularly those targeting adolescents from lower socioeconomic background who are most at-risk of inactivity [19], and most trials targeting adolescents have not been evaluated using whole day objective PA measures [17, 18]. The implementation of comprehensive school PA programs and the use of the Health Promoting Schools (HPS) framework for school based programs can enhance PA outcomes [13, 20, 21], with systematic review evidence supporting the effectiveness of interventions that address: the curriculum (physical education (PE) and sport) [22]; the school environment (e.g. recess and lunch activities and equipment) [23]; and the broader school community (partnerships with community PA providers and parents) [24].

Our recent cluster randomized controlled trial ‘Physical Activity 4 Everyone (PA4E1)’, conducted in ten (5 intervention) secondary schools in socio-economically disadvantaged areas in Australia, demonstrated an increase in adolescent daily minutes of MVPA and a smaller increase in unhealthy weight gain over two years [15, 25]. Furthermore, the PA4E1 program delivered these improved health outcomes at a low incremental cost [15, 26]. Briefly, the PA4E1 efficacy trial consisted of seven PA practices including increased PA within physical education (PE), development of student personal PA plans, enhanced school sport programs, recess and lunchtime PA, school PA policy, links with community PA providers and links with parents. Secondary schools were supported to adopt the seven PA practices via six implementation support strategies including location of a change agent within the school one day per week.

In order to improve adolescent PA levels, the implementation at-scale of effective programs and practices, such as PA4E1, is required to achieve population level health benefits [27]. However, a review of PA interventions that have been scaled-up (fifty or more sites), identified that only six of the 16 interventions were within the school setting, and none within the secondary school setting [28]. Furthermore, a review of implementation trials [29] conducted within the school setting identified that of the 27 trials included in the review, only six interventions focused on school-based PA and had evaluated the effectiveness of methods to improve the implementation of evidence based policies, programs or practices. None of these school-based PA implementation trials had been conducted within the secondary school setting. The review concluded that whilst modest improvements in implementation appeared possible, the quality of evidence was poor and the characteristics and cost of effective implementation strategies remains relatively unknown [29]. Identifying the barriers or enablers to program implementation via formative evaluation and consultation with key stakeholders, co-development of contextually-relevant implementation support strategies and the use of theories and/ or frameworks to guide intervention design were recommended to be incorporated into future trials to maximize the likelihood of the effective of implementation.

To address this gap in the evidence base, the aim of this study is to assess the effectiveness of a multi-component implementation support strategy to improve implementation, at-scale, of evidence-based school PA practices of the PA4E1 program within socio-economically disadvantaged secondary schools, compared to usual school practice. The impact of the intervention on student PA levels (overall and school day MVPA) and adiposity will be assessed as secondary outcomes, in addition to the cost and cost effectiveness of implementation.

## Methods/Design

### Study design

**Fig. 1:**
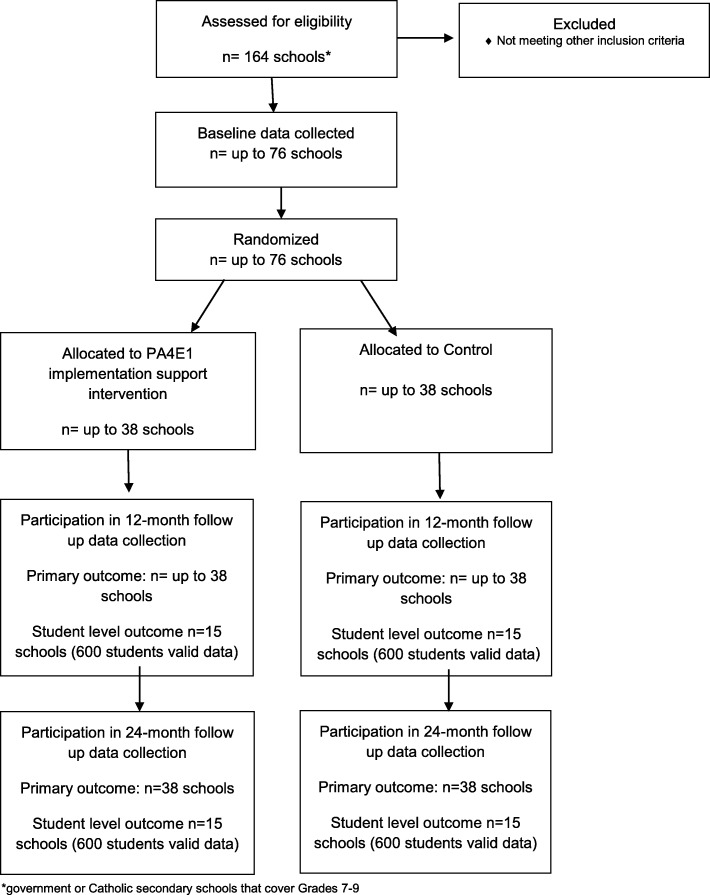
Consort Flow Diagram

This study will employ a cluster randomised controlled design (Fig. 1). A type III hybrid implementation-effectiveness trial will be conducted combining both implementation outcomes and individual student level PA and anthropometric outcomes [30].

The research will be conducted and reported in accordance with the requirements of the Consolidated Standards of Reporting Trials (CONSORT) Statement [31, 32] and the Standards for Reporting Implementation Studies (StaRI) Statement [33]. Secondary schools will be randomised to receive either the multi-component implementation support intervention, or to a control arm (usual school practices) after baseline data collection. Assessment of school PA practices will occur at baseline, 12 months (mid intervention) and 24 months. A nested study involving 15 schools per arm will be used to evaluate student level outcomes, objectively measured student PA via accelerometers (overall minutes of MVPA per day, and minutes of MVPA within school hours) and indicators of adiposity (weight, Body Mass Index (BMI)), measured at 12 and 24 months (Fig. 1). Approval will be obtained from relevant ethics committees. The trial has been prospectively registered ACTRN12617000681358.

### Setting

The study will be conducted across four local health districts in the state of NSW, Australia (Hunter New England (HNE), South Western Sydney (SWS), Central Coast (CC) and Mid North Coast (MNC)). These districts are geographically widespread and encompass major city (SWS, CC, HNE) and regional/rural areas (HNE,MNC, CC) [34].

### Sample and participants

#### Schools

Secondary schools in NSW cater for students aged 12 to 18 years old (Grades 7–12) and operate over four terms per calendar year. Schools are required to ensure students undertake 300 h of Health and PE each year, from Grades 7 to 10 [15]. Schools should also provide opportunities for students to engage in PA through school sport, PE and other associated informal opportunities in line with NSW Department of Education policy; to provide a minimum of 150 min of planned moderate intensity PA, with some vigorous intensity, to students each week in government schools [35].

Secondary schools within the study regions that meet the following criteria will be eligible to participate in the study: (1) Government and Catholic schools; (2) enrol students in Grades 7–9; (3) located in areas classified as being disadvantaged by the SEIFA Index of Relative Socio-economic Disadvantage (suburb in lower 50% of NSW) [36]; (4) not fully selective/sports/performing arts/agriculture/boarding schools; (5) not participating in other major whole school PA trials or initiatives. Eligibility against the first four criteria will be determined from publicly available data [37], and for the remaining criteria, based on contact with schools during recruitment. The study will recruit up to 76 schools. A nested sample of 30 schools will be selected for student level data. The nested sample (*n* = 30) will include co-educational schools only and will be randomly selected from the main study sample.

#### PE teachers

At baseline, 12- and 24-months, Head and all PE teachers within each school will be invited to participate in the study assessments.

#### Students

All students within Grade 8 (12 month follow-up) and Grade 9 (24 month follow-up) from the schools in the nested sample (n = 30) will be invited to participate in study measures. Classes catering for students with severe physical or mental disabilities will be excluded from eligibility for collection of student measures.

### Recruitment procedures

#### Schools

Prior to recruitment, the study will be promoted to school sector Regional Directors within the NSW Department of Education (DoE) and the relevant Catholic school dioceses, and to Government school staff in relevant regions through existing electronic communication systems (e.g. email). A list of schools deemed eligible and within strata (based on local health district (four) and school sector (two) will be assembled). The list of schools will be randomly ordered within their strata using a random number function in Microsoft Excel by a statistician not involved in contacting schools or in the study delivery [15]. A letter inviting schools will be emailed to all Principals, requesting the information be shared with the Head PE teacher. The Principal and/ or Head PE teacher will be contacted by telephone by a dedicated recruitment project officer with PE teacher training. A face-to-face or telephone meeting will be offered to outline the requirements of the study and confirm eligibility.

#### Teachers

Following school consent, all PE staff will be provided with a study information letter that outlines the purpose of the study and level of involvement expected.

#### Students (nested sub study)

Parents and students will be provided with letters outlining the study and a student consent form. The form will allow consent to be provided separately for participation in accelerometry, anthropometric measures, and student surveys. Parents will be provided with a telephone number to leave a message if they do not want to be prompted about consent or do not want their child to participate in study measurements. Parents who have not returned a consent form or left a message within two weeks will be telephoned by staff employed through the education sector who will explain the study and seek verbal consent using a standardised script [15].

As some schools will have Aboriginal student enrolments of 10% or greater [37], the study will adopt strategies to facilitate involvement of Aboriginal parents and students (with Principal permission) including: information on study letters on how to make contact with an Aboriginal member of the research team; contact with a school Aboriginal staff member to make them aware of the study and seek advice on ways the schools would usually communicate with parents; offer information to Aboriginal Education Consultative Groups (AECGs) through school channels; and having an Aboriginal staff member present on data collection days. As some schools will have high proportions of students with parents who speak languages other than English (LOTE) at home [37], the study will adopt strategies to facilitate involvement of such parents and students, including (with Principal’s permission): strategies the schools would usually use to communicate with such parents, including translation of study letters and consent forms into the major languages; adapting telephone prompting protocols to allow involvement of interpreters or school staff; and having appropriate staff members present at data collection sessions.

### Randomisation

Consenting schools will be randomised to either program or control condition using a random number function in Microsoft Excel. Block randomisation (1:1) will be undertaken within the strata (four local health districts and two school sectors). Principals will be notified of their school allocation following baseline collection of school practice measures. Schools consenting to student level data (nested study) will be randomly selected within strata using a random number generator in Excel, and at stratum sizes proportional to that observed in the main study. Schools will be notified of student level measurement involvement when notified of group allocation.

### Blinding

Due to the inability to blind schools and teachers to the program strategies, the study will be conducted as an open trial. Data collectors will not be informed of group allocation, however blinding cannot be assured as this may be disclosed by school staff or students. The statistician undertaking data analysis will be blinded to study group, by using treatment numerical codes only.

### Program group

#### Theoretical framework

The Health Promoting Schools (HPS) Framework and the Social Ecological Theory underpin the seven evidence-based school PA practices used in the initial efficacious PA4E1 trial [15] and in the current trial. The Behaviour Change Wheel [39] and the Theoretical Domains Framework [40] were used to develop the multi-component implementation support strategies.

#### School physical activity promotion practices

Table 1 provides an overview of the school PA practices that are the focus for the current trial. The expectation is that schools use one school term for planning, and then will commence practice implementation in the second term with a focus on incoming Grade 7 students (age 12–13). Practice implementation builds over two school years (8 school terms) and is designed to be ongoing.

**Table 1:** Overview of the evidence based intervention (PA4E1 PA practices) including standards required of program schools

Physical activity practices by Health Promoting Schools domain	
Curriculum, teaching and learning	
1. Quality PE lessons: PE department uses documented principles or guidelines for teachers to maximize PE quality, active learning time and student engagement in PE lessons (Program schools to use the SAAFE principles- Supportive, Autonomous, Active, Fair, Enjoyable [41]). Each PE teacher to have peer observation of a practical PE lesson, at least once a year^a^. *Desirable – peer observation feedback is against the department’s quality PE principles.*	
2. Student PA plans: Process in place for all students to set personal PA plans in Grade 7, and preferably for more grades 7–10^b^. Plans/process to i) include personal goals to improve or maintain activity or fitness ii) include actions and timelines to achieve goals and iii) include progress monitoring and goal review at least once within the year.	
3. Enhanced school sport program: scheduled for delivery to all students in at least one Grade, between 7 and 10. A structured PA Program to be: short duration (10–12 weeks), designed to improve adolescents’ fitness and provide them with knowledge, motivation and skills to engage in a range of lifelong physical activities, includes directed practical PA sessions (Program schools to use the Resistance Training for Teens program for all Grade 7 [46]. This program includes: an interactive student seminar; structured PA program focused on muscular fitness and lifelong activities, and a smartphone app.	
Ethos and environment	
4. Recess/ lunchtime physical activity: Supervised recess and/or lunchtime PA sessions offered to all students in Grades 7–10 at least 3 days per week. PA equipment freely available to students at least 3 days per week at recess and/or lunch. *Desirable at least one organized recess or lunch activity per week targeting girls. Sessions promoted to students at least once per term.*	
5. School Physical Activity Policy or Procedure: inclusive of the following: school provides all students in Grades 7–10 with at least 150 min/week of MVPA during school time; school implements supportive practices to enhance all students’ PA (at least 3 of practices 1–4, 6–7 in this table)^c^.	
Partnerships and services	
6. Links with community physical activity providers: Links with community PA providers- Schools promote and engage with community based PA providers to support ‘outside of school time’ activity. At least three links go beyond promotion of the provider (e.g. in newsletters) to involve an agreement, connection, partnership or engagement (e.g. out of hours sessions on school facilities, presentation by providers at school). Links communicated to students and families at least once per term^d^. *Desirable - at least one link to not carry a cost/or be low cost in an ongoing way.*	
7. Communicating physical activity messages to all parents of students in Grades 7–10 at least once per term (excludes messages only about school events e.g. carnivals, or school sports timetables or results, or promotion (advertisements for) community PA providers)^d^.	

^a^Program schools were asked to aim for peer observations once a semester

^b^Program schools asked to set plans for Grade 7 at 12 months, 7 and 8 at 24 months. For outcome assessment schools reporting plans developed for at least Year 7 was considered sufficient

^c^Program schools asked to include practices 1–4, 6 and 7 in their policy

^d^Program schools asked to use multiple modes to promote community links and to communicate PA information to parents (eg newsletters, parent app, parent information evening)

#### Implementation support intervention

The multi-component implementation support intervention will consist of seven strategies delivered over two school years (Table 2). As outlined below, the implementation support intervention was developed for the original trial, then adapted for scale-up for this study.

**Table 2:** Overview of the multi-component implementation support intervention

Implementation support strategies (implemented over 8 school terms)	
1. Obtaining executive and leadership support: School executive sign a partnership agreement outlining commitment to the program. School team/committee formed (new team or re-alignment of existing team, inclusive of in-School Champion and school executive member) to oversee program, meet at least once per term.	
2. Embedded school staff: in-School Champion: An existing school PE teacher is allocated a half day per week funding to support program implementation (funding of $350AUD a fortnight provided by the Department of Health to cover half–day a week release for the duration of the intervention (8 school terms, which is 2 years)).	
3. External implementation support: A Support Officer (health promotion officer, ideally with PE teacher training) co-located within the relevant local health district will make regular contact (weekly for 12 months, and as needed for the remaining 12 months) with in-School Champion via phone, email and/or face-to-face site visits.	
4. Teacher professional learning: Accredited with teacher accreditation body NESA [38] • In-School Champion training – Two × 1-day face to face training sessions - hosted by PA4E1 implementation team in Term 1 and Term 5. These involve paid overnight accommodation, all meals and transport costs for those in-School Champions that attend • Quality PE training – 8 × 10-min online training videos delivered via the password protected program website focused on the SAAFE principles to be viewed by all PE teachers and discussed as a PE department, including some brief quiz questions relating to the videos • Enhanced school sport training – PA4E1 in-School Champion (or other teachers involved in delivering the program could attend 1 day face-to-face training offered through NSW Department of Education (School Sport Unit). – Resistance Training for Teens (course costs paid by project for in-School Champion), or equivalent training run by PA4E1 implementation team - Term 1–2 (not accredited). Other teachers can attend (not paid for by project) • School PA policy training – in-School Champion to undertake existing online school PA policy training run by the NSW Department of Education (Government schools only) [42]	
5. Resources: Primarily distributed via the program website (PA4E1 Online) accessed by in-School Champion and other PE teachers as required: • Resources on the program website include: Overview of program presentation (Powerpoint presentation), project milestones to be achieved each term (over 8 terms, with term 1 as a planning term - this includes preparation, training and practice implementation milestones), online quality PE training (SAAFE Principle videos (6 videos - one overview and one per Principle) and worksheet, peer observation materials), student personal activity plan templates, recess and lunch resources, policy templates, examples of community PA providers, tips and frequently asked questions • Some PA equipment provided to support the delivery of recess and lunchtime PA ($100AUD equipment voucher) and enhanced schools sport program (5 Gymsticks/school) • Posters outlining Quality PE principles (SAAFE Principles [41] to be displayed in PE department by 12 months	
6. Provision of prompts and reminders: emails or phone calls (occurring weekly in the first 12 months and as needed in the second 12 months from Support Officer encouraging schools to implement PA practices, complete training. Automated messages sent each term via the program website to in-School Champions about completion of teacher professional learning and completion on online termly surveys.	
7. Implementation performance monitoring and feedback – termly performance monitoring surveys are completed by in-School Champions via the program website, and a feedback report on progress against milestones for each PA practice is automatically generated and sent to the school Principal, Head PE teacher and in-School Champion. The report is used by the external Support Officer to identify practices that can be achieved as ‘easy-wins’ (i.e. need only a little bit more effort to be achieved) and to guide implementation support and overcome barriers to implementing PA4E1 more broadly.	

#### Adaptation of the PA4E1 program school practices and implementation support intervention for implementation at scale

For this study, minor adaptations were made to the school physical activity promoting practices to address barriers raised regarding their implementation identified in the original trial. The multi-component implementation support strategies were similarly adapted to address barriers to their delivery and utility in the original trial, and to aid their implementation at-scale. The adaptations were made based on a review of existing models and factors for scaling up public health interventions [43] and a scoping review of frameworks for adapting public health interventions [44].

The key steps for adapting the implementation support strategies were undertaken iteratively, and involved: i) identifying barriers and enablers to change based on a review of the literature, focus groups with PE teachers and students, key informant interviews with school executives participating in the initial trial and Aboriginal stakeholder meetings and; ii) using the Theoretical Domains Framework (TDF) [40] and Behaviour Change Wheel (BCW) [39] to map the identified barriers to school implementation of PA practices and to evidence based implementation support strategies (behaviour change techniques); iii) revising the PA practices and implementation support strategies and delivery modes based on scalability (using the APPEASE criteria [45]) and iv review of the resulting adaptations by expert researchers and practitioners.

The main adaptations to the school physical activity promotion practices were: a broader focus on quality PE [41] rather than solely on ‘active PE’; and the use of a modified enhanced school sport program developed in partnership with the NSW Department of Education, designed to meet the needs of schools and supported by the Education system known as ‘Resistance Training for Teens’ [46] (rather than Program X [16]). These adaptations were made to support delivery at scale and synchronise with existing systems.

Adaptations to the multi-component implementation support intervention strategies were: 1) embedding school staff by appointing and funding an in-School Champion along with access to a local health promotion Support Officer to replace the external change agent model used in the original trial; 2) a change in the modality of teacher professional learning from face-to-face training to online where possible, and use of accredited education sector courses where possible rather than project specific learning programs, to allow teachers to include attendance at these courses within their professional accreditation [38]; 3) the provision of tools (such as PE observation forms) and resources via a program website rather than paper-based; 4) prompts and reminders and performance monitoring and feedback automated to each school (via completion of termly progress surveys) to occur via the program website rather than verbal/email prompts and manually generated feedback reports.

Additional file 1: Table S1 presents an overview of the identified barriers to school implementation and scale-up, the mapping of these barriers to the TDF [40] and BCW [39], the behaviour change techniques selected to address the barriers, and a further description of how these have been incorporated into the multi-component implementation support intervention used in the current trial.

### Control group

Control schools will participate in study measures and continue delivering usual PE lessons, school sport and other PA programs and practices. PA4E1 program materials, inclusive of access to the program website will be available to these schools on study completion.

### Data collection procedures and measures

An overview of data collection is shown in Table 3. The primary outcome will be the proportion of schools in each group implementing any four of the seven PA practices (the prior trial showed a significant effect on student MVPA at 12 month mid-point [47], by which time schools had implemented 4 of the 7 practices). Secondary outcomes will be the mean number of PA practices achieved, whether or not schools implement each of the seven PA practices (to determine compliance with each practice), as well as student level outcomes (objectively measured PA and adiposity indicators). Economic outcomes will include: 1) the direct cost of the multi-component implementation support intervention from multiple stakeholder perspectives; 2) average and incremental cost-effectiveness ratios, subject to assessment of effectiveness, and 3) budget impact calculated from a public finance perspective.

**Table 3:**
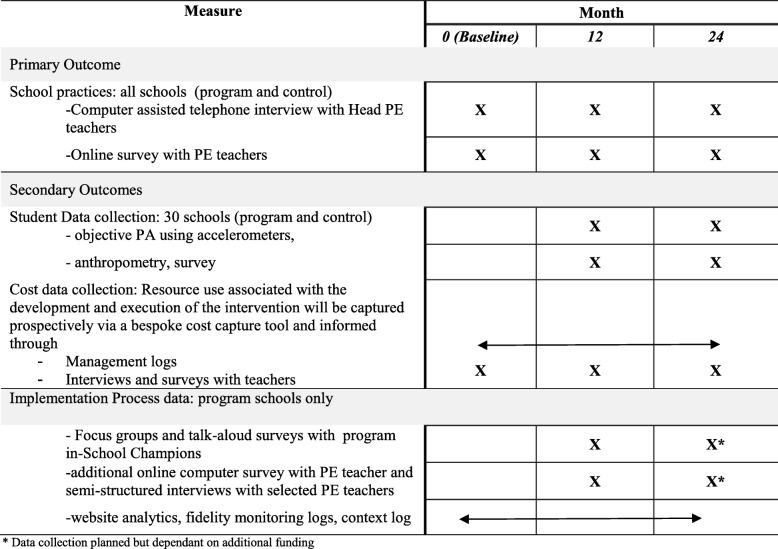
Schedule of study data collection and measures

^a^Data collection planned but dependant on additional funding

A separate process evaluation protocol will be developed and published elsewhere.

#### School characteristics/ Head PE teacher characteristics

Publicly available data [37] will provide information on school sector, postcode, size (total enrolments), Indigenous enrolments, and students who speak a LOTE at home enrolment for all schools approached for consent. For schools participating in the project the following characteristics will be obtained through a Head PE teacher computer assisted telephone interview: number of PE teachers and full time equivalent PE positions; language groups most commonly represented when the school has more than 10% of LOTE students.

Computer assisted telephone interviews with Head PE teachers and online surveys with all PE teachers conducted at baseline, 12 and 24 months, will ask for background information including sex, PE training, years of teaching experience, how long they’ve taught PE at their current school, and grades they teach in the current year.

#### School practices

Measures of the seven PA practices shown in Table 1 will be undertaken via the computer assisted telephone interviews with Head PE teachers, administered by trained interviewers. The items will be adapted from those used to collect process data in the previous PA4E1 trial [15, 25, 47] and will be forwarded by email to participants prior to the interview.

#### Student level variables (nested sub study)

*Physical activity:* Whole-day objective PA measurement will be undertaken using wrist worn Actigraph GT9X-BT or GT3X+ accelerometers [48], distributed to students at school within class, at the same time as students’ complete anthropometric measures and surveys. Accelerometers have displayed acceptable intra- and inter-instrumental reliability and provide a valid and reliable estimate of PA in young people [48, 49]. Students will be asked by trained research assistants to wear the accelerometers on their non-dominant wrist during waking hours for seven consecutive days. To improve compliance, student and/ or parent mobile phone numbers will be requested via the consent form, and a text message sent by the researchers each morning reminding students to wear the accelerometer [15]. School start and finish times that apply during the assessment period will be requested from schools.

#### Anthropometric data

Research assistants will be trained in measuring height and weight (used to calculate body mass index; BMI) using the International Society for the Advancement of Kinanthropometry (ISAK) procedures [50]. Where possible, all measurements will be taken in the morning [51]. Weight will be measured in light clothing without shoes using a portable digital scale (Model no. UC-321PC, A&D Company Ltd., Tokyo Japan) to the nearest 0.1 kg. Height will be recorded to the nearest 0.1 cm using a portable stadiometer (Model no. PE087, Mentone Educational Centre, Australia). The physical assessments will be conducted in a sensitive manner, with student measurements taken behind a privacy screen. Body mass index (BMI) will be calculated as weight/height squared (kg/m^2^). Weight status will be determined using International Obesity Taskforce definitions [52].

#### Survey

The student survey, which will take approximately 20 min, will be undertaken to assess: student socio-demographic characteristics (age, sex, Aboriginal or Torres Strait Islander status, languages spoken at home, postcode of residence); which years they have attended the school; self-reported PA [53]; school PA behaviour (items from Questionnaire Assessing School Physical Activity Environment Q-SPACE [54]); autonomous motivation for PA (intrinsic motivation and identified regulation subscales of the Behavioural Regulation in Exercise Questionnaire 2 (BREQ-2) [55]), social support for PA [56]; perceptions of school PA environment (items from or adapted from scales including Q- SPACE [54], Student Engagement in School Questionnaire [57], Teacher as Social Context questionnaire [58] controlling teacher scale [59]; and general health and well-being (KIDSCREEN 10 [60]). The survey will be completed by students at 12 and 24-months via a tablet provided by the research team.

#### Cost data

Cost data pertaining to the development and implementation of the intervention will be collected prospectively using a resource use capture tool. The tool, developed in MS Excel (2013), allows the input of resource use data from each of the stakeholders involved in implementing the intervention for the following cost categories: labour (health service), labour (non-health service), materials, joint costs and miscellaneous costs. Management logs routinely used by the Support Officers will inform the identification and measurement of resource use associated with the intervention. Interviews and surveys with teachers will also inform the identification and measurement of resource use from the perspective of the schools.

#### Implementation process outcomes

A full process evaluation protocol, with detailed aims, will be published separately. Briefly, the evaluation of implementation outcomes for the trial will be aligned to the assessment of domains recommended by Proctor et al. 2011 [61] including appropriateness, acceptability, adoption, feasibility, fidelity, penetration (reach) and sustainability. In addition, usability and engagement with the program website will be investigated [62]. A mixed-methods assessment will use data obtained from a variety of sources including PE Teacher and in-School Champion termly implementation surveys from program schools (Table 3). Appropriateness, acceptability and feasibility will be assessed using validated survey items developed by Wiener et al. [63]. Adoption (primary trial outcome) will be assessed via computer assisted telephone interviews with Head PE teachers. Fidelity and dosage (and context) will be assessed via a fidelity monitoring log [64] and a project specific fidelity checklist. Reach and sustainability will be assessed using items developed by the project team, specific to the program. Qualitative data sources will include in-School Champion focus groups and semi-structured interviews with PE teachers, as well as talk-aloud surveys relating to the engagement, usability and user experience with the program website.

The online surveys of PE teachers in program and control schools will provide data on practice barriers (Table 3).

#### Supporting schools with data collection

All schools will be offered half a day of teacher relief ($175AUD) at each practice data collection point (baseline, mid-point and follow-up) to reimburse the school for their time in assisting with data collection. In addition, schools participating in student data collection (nested sub study) will be offered 1.5 days teacher relief ($525AUD) at each of the 12 and 24 month collections, plus additional funding if school staff are involved in parent consent gaining calls. This will allow a school staff member to be present during the student data collection sessions. Prior to data collection days, a list of students with parental consent will be provided to the school so schools can consider whether additional research staff may be required to assist students with limited literacy.

### Statistical analyses

School characteristics will be summarised separately for schools participating in the trial and those refusing participation, and for program and control schools. Student characteristics (nested sample) will also be summarised for each group for the 12 month (Grade 8) and 24 month (Grade 9) samples.

#### Primary outcomes

The primary outcome is the proportion of schools implementing at least four of the seven PA practices [47] based on the Head PE teacher computer assisted telephone interview data. The analysis will follow intention-to-treat principles, with multiple imputation the primary method of dealing with missing data. Per protocol analyses will also be performed, based on those schools receiving the full implementation support intervention.

Differences between treatment groups in the primary outcome at 12 and 24-months will be assessed using generalised linear regression models (a separate analysis conducted for each time point). A log link and binomial distribution function will be used (a logistic link will be utilised if the analysis model fails convergence). The stratification variables will be included in the models as covariates.

#### Secondary outcomes

##### School practices

Secondary outcomes will include the mean number of PA practices achieved and the proportion of schools meeting each of the practices (Table 1). Analysis will be undertaken similarly to the primary outcome, with a normal distribution function for the mean number of practices outcome.

##### Student level measures (nested sub study)

Student data will be analysed if accelerometers are worn for ≥480 min per day on ≥3 days [65]. Appropriate cut-points based on emerging evidence and expert advice for wrist worn accelerometers will be used to categorize different intensities of PA [66]. Analysis will use all available data and follow the intention to treat principle. Analysis of minutes of MVPA per day (overall, within school hours as determined by school timetables) at each of the two follow-up time-points (corresponding to 12 and 24 month follow-up) will be undertaken using linear mixed models, including school level random intercept to model the clustering (30 clusters) of students within schools, and a random intercept for individuals. Analyses will adjust for demographic characteristics (sex, age, Aboriginal and Torres Strait Islander background). Least square mean differences between intervention and control students will be presented at both time points, together with 95% confidence intervals and *p*-values. A similar analysis will be undertaken for anthropometry outcomes, weight, BMI, BMI z scores. Exploratory subgroup analyses will be undertaken by sex, Aboriginality and LOTE.

### Economic analyses

The economic evaluation will be performed as a within-trial analysis, indicating that only costs and effects that accumulate within the trial duration are included. In this study, the prospective, trial-based economic evaluation will involve (i) resource use modelling to describe the investment required to develop and execute the implementation-intervention from all relevant stakeholder perspectives; (ii) a cost consequence analysis to identify, measure and value all associated costs and outcomes associated with the program compared to usual practice and (iii) subject to assessment of effectiveness, a cost-effectiveness analysis based on both primary school level outcome and secondary, student level outcomes.

#### Costs

The cost model will be based on the concept of opportunity cost, that is, the value of the benefit forgone in not employing a resource in a different use. In this analysis, market prices will be used as a proxy for this value. Labour costs will include an overhead to allow for additional costs of employment. Materials refer to non-abour cost items such as stationary (education materials) or electronic hardware or software. Joint costs are defined as those costs incurred in connection with multiple projects. For example, the maintenance costs of a website portal supporting different interventions. Capital costs refer to major one-off investments such as the purchase of additional office buildings or motor vehicles. Miscellaneous costs are considered to be those costs not easily classified into the other categories. The primary outcome from the cost model will be an estimate of the mean cost per school of implementing the intervention. Costs for each of the intervention elements will be reported separately and jointly. The analysis will adhere to cost and economic analysis guidelines [67–69]. To maintain a conservative approach to cost estimation, the non-capital implementation costs are not amortised.

#### Cost consequence analysis

Cost consequence analysis, the listing of all cost/benefit implications of each alternative, represents the simplest form of economic evaluation. In this analysis, where the collection of outcomes is diverse, it has value in being able to inform spending decisions by capturing the range of returns generated by the investment.

#### Cost effectiveness analysis

Subject to assessment of effectiveness, the trial-based evaluation will include assessment of cost-effectiveness. It has been suggested that one of the ways to improve efficiency in conducting economic evaluations of implementation interventions, is to confine the study to measures of the care process or intermediate outcomes [70]. This is the approach adopted in this study, where the cost-effectiveness outcomes, the incremental cost effectiveness ratios (ICER) will be: (i) incremental cost per percent change in the proportion of schools implementing at least four of the seven PA practices, reflective of the primary trial outcome and (ii) incremental cost per unit changes in student-level outcomes (minutes of MVPA per day, % reduction in BMI and metabolic equivalent (MET) hours gained). The ICERs will be calculated as the arithmetic mean difference in cost between the intervention and usual practice arms divided by the arithmetic mean difference in effect [68, 71].

Uncertainty analysis will be conducted using parametric and non-parametric bootstrapping techniques and sensitivity analyses will be conducted to explore the robustness of the economic outcomes to any uncertainty associated with key parameters.

#### Budget impact analysis

A budget impact analysis considers specific requirements of policy makers taking account of local perspective and time horizons. Subject to assessment of effectiveness, the results of the cost and cost-consequence analyses will be reconfigured as a budget impact statement reflecting the costs and outcomes falling directly within specific budgets and within the usual timeframe of the budget holder.

All of the analyses will be constructed in Microsoft Excel and will adhere to international reporting standards for economic evaluations [67].

#### Process data

A mixed methods process evaluation based on Proctor et al. [61] will be undertaken using a descriptive analysis that will be detailed in a separate protocol.

### Sample size

Based on publicly available data there are approximately 120 potentially eligible schools. Based on school consent rates in previous trials conducted by the researchers of around 65–70% [15], a sample of 76 schools (38 per arm) will provide 80% power to detect an absolute increase of ~ 35% between groups in the proportion of schools implementing at least 4 of the 7 items at 12 and 24-months (alpha 0.025 to allow for program effect at 12 or 24 months). Without prior data on baseline levels of school practices, this calculation makes the conservative assumption that 40% of schools in the usual care arm achieve this target at follow-up.

For student level variables it is estimated that a nested sample of 30 schools and a student consent rate of 65% will yield about 1200 students (600 per group) with valid accelerometer data. Using an ICC of 0.03 [15] and a standard deviation of 23 min of MVPA, will allow a 6.2 min [47] difference in the mean difference between groups at each separate follow-up to be detected with 80% power and a 2.5% type 1 error rate.

### Trial discontinuation or modification

It is not anticipated that any events would occur that would warrant discontinuing the trial. Any unforeseen adverse events will be reported to the Hunter New England Human Research Ethics Committee (primary approval committee) and advice sought regarding required action. The trial registration record will be updated with any protocol modifications and any deviations from original protocol will be reported in study outcome papers.

## Discussion

Implementation research is a neglected area of scientific investigation, particularly within community settings [72]. Research evaluating methods for increasing implementation of effective health policies, programs or practices represents just 1% of health promotion research publications. [73] Similarly, recent systematic reviews have identified few implementation intervention trials across a variety of community settings and health behaviours, and a scarce number of implementation trials conducted at-scale [29, 72, 74]. Rigorous trials of theoretically based implementation interventions provide the best evidence to identify solutions to bridge this evidence-practice gap and assist in providing critical information needed to support implementation at scale. Scaling up effective public health interventions is the ultimate goal, as unless interventions are delivered at scale, they offer little benefit to population health [75]. This trial is one of few large-scale, randomised trials of an intervention to improve PA practice implementation at-scale in a non-clinical setting. Using a systematic, theoretically grounded approach, this trial will provide critical information to develop more efficient and effective next generation trials focused on implementation scaling-up and scaling-out.

This will be the first implementation study at-scale of a PA intervention targeting PA practices in socio-economically disadvantaged secondary schools. The findings will inform future efforts to support schools to implement health promoting policies and programs. Further, the findings could provide a basis for supporting implementation of other evidence based policies in this setting.

## Additional file

Additional file 1: **Table S1.** Description of identified barriers to implementing the evidence based intervention mapped to the Theoretical Domains Framework and Behaviour Change Wheel’s COM-B and the behaviour change techniques and description of the implementation support intervention. (DOCX 23 kb)

## Abbreviations

BMI: Body Mass Index
HPS: Health Promoting Schools
LOTE: Language other than English
MVPA: moderate-to-vigorous intensity physical activity
NSW: New South Wales
PA: Physical Activity
PA4E1: Physical Activity 4 Everyone
PE: Physical Education
