# Supplementary material for: A cluster randomised trial of an intervention to increase the implementation of physical activity practices in secondary schools: study protocol for scaling up the Physical Activity 4 Everyone (PA4E1) program
Source: BMC Public Health. 2019 Jul 4;19:883. doi: 10.1186/s12889-019-6965-0 (PMC6610944; doi:10.1186/s12889-019-6965-0)
Supplement: Supplementary file 1 — Table S1. Description of identified barriers to implementing the evidence based intervention mapped to the Theoretical Domains Framework and Behaviour Change Wheel’s COM-B and the behaviour change techniques and description of the implementation support intervention. (DOCX 23 kb) [file 12889_2019_6965_MOESM1_ESM.docx]

**Supplementary Table 1.** Description of identified barriers to implementing the evidence based intervention mapped to the Theoretical Domains Framework and Behaviour Change Wheel’s COM-B and the behaviour change techniques and description of the implementation support intervention

| **Physical activity practice** | **Barrier**  **Identified** | **TDF (COM- B)** | **BCT** | **Detailed explanation of Implementation support strategy (including mode)** |
| --- | --- | --- | --- | --- |
| General delivery of PA4E1 (multicomponent PA programs in schools) | - Executive support to prioritise PA within the school - Time dedicated to making changes due to busy school environment - Knowledge of the link between PA and academic achievement - Motivation and skills to support the change process within the school | Executive function  Motivation | - Executive support - Provide knowledge - Persuasion - Financial incentive - Audit and feedback | - Health promotion school Support Officers will meet with Principals and Head PE teachers to communicate holistic benefits of PA including for school engagement, and educational outcomes (imparting knowledge, persuasion).   School Principal to present about program at a whole of staff meeting and to support formation of a committee to support program implementation   - Financial incentive for schools to release a PE teacher to be School Champion. The in-School Champion will lead the implementation of practices - School will have access to an external Support Officer, who will contact the school regularly (weekly via email, phone or face-to-face) to assist the in-School Champion to implement the 7 school PA practices and.to overcome barriers to support schools - In-School Champions will receive 2 x face-to-face training sessions to support implementation of PA4E1 - Audit and performance feedback will occur at the end of each school term (x8) via the program website, with feedback sent to school Principal, Head PE teacher and in-School Champion. Feedback reports will be used to guide the support provided to the school by the external Support Officer. |
| High quality and active PE | - Knowledge of quality PE principles - Skills to deliver high quality and active PE - Teacher motivation to change practice - Role identity - Remembering to put practices into place amongst a busy school environment | - Knowledge - Skills - Belief about capabilities - Motivation and goals - Memory, attention, decision making process | - Information about behaviour/ outcome - Modelling behaviour by others - Graded tasks - Rewards; incentives - Prompts, triggers and cues | - All PE staff will receive training (8 x 10 minute modules, delivered via the program website to maximise reach and minimise cost) on quality PE principles and strategies to maximise active learning time (SAAFE) ([1](#_ENREF_1)). Online modules will role model teachers delivering high quality PE lessons and will incorporate graded delivery. PE teachers will set a goal of completing 2 modules/term over the first 12 months of PA4E1. - Each module will include a motivating explanation of why the practice is important, an overview of the evidence base and their role in achieving high quality practice presented by an international expert and leader in PE practice (Author DL). In addition, strategies that can be incorporated into lessons, and examples of good and not so good practice (motivation, role modelling, role identity) will be incorporated. Teachers will be requested to complete a short knowledge questionnaire following the online training module. Training will be accredited allowing the professional learning hours to contribute towards professional accreditation. - The in-School Champion will prompt PE teachers to complete the online training, and remind teachers to undertake peer observation (encouraged) and provide feedback to colleagues. Additionally, each PE department will receive a set of posters to be displayed in the staff meeting room which can be used to remind teachers of the quality PE principles. |
| Student personal PA plans | - Time to work with students to develop a plan - Flexible delivery in schools to fit within existing units of work | - Action planning - Skills - Memory, attention | - Increasing skills: problem-solving, decision-making, goal-setting - Prompts, triggers and cues | - School Champions and PE teachers will be provided with a personal PA plan template and detailed instructions on how the strategy can be adapted to the schools needs and used with students including a rationale for the personal PA plan, links with school curriculum outcomes, and instructions on how to complete and review the plan. - PE teachers will be prompted to complete the action with their classes via the in-School Champion. |
| Enhanced School Sport program focused on resistance training | - Lack of skills to deliver the program - Timetable does not allow time to deliver - Student choice to participate - Program flexibility to adapt to different schools needs and resources | - Skills - Action planning - Motivation and goals - Environment context and resources | - Increasing skills: problem-solving, decision-making, goal-setting - Planning, implementation - Rewards; incentives Environmental changes | - In-Schools Champions and teachers responsible for delivering will be linked into an existing professional learning program delivered via the NSW Education system and available to all schools. - Two teachers from each school will be provided funding to attend the training course. - Attendance at the course will result in 2 hours of accredited teacher professional learning. Following attendance at the course, schools will be supported to timetable the delivery of the course into existing teaching schedules (either via school sport or through other curriculum avenues) which allow all year 7 students to participate in the enhanced sport program. - Once the program is scheduled, schools will receive 5 x gymsticks as an incentive. |
| School PA policy | - Lack of executive support to develop a school PA policy - Lack of skills on how to develop a whole school PA policy - Time to develop and ratify policy | - Social/Professional role and identity - Knowledge and skills - Motivation and goals | - Social processes of encouragement, pressure and support - Increasing skills: training - Rewards; incentives (incl. self-evaluation) | - In-School Champions will be supported to attend an existing online training course delivered by the NSW Education system. - The online course supports schools by imparting the knowledge and skills needed to develop a whole of school policy and procedure. - In addition, a policy template will be developed consisting of each of the 7 PA practices to enable the schools to model a policy document. - Attendance at the accredited course will allow the professional learning hours to contribute towards professional accreditation. - The policy will be signed off by the school Principal. |
| Recess and Lunchtime PA | - Limited teacher supervision (attitude of having to give up lunch beaks) - Teacher motivation to schedule organised activity/need for additional teachers on duty - Limited available equipment - Students remembering and being motivated to participate | - Social/Professional role and identity - Action planning - Environmental restructuring - Memory, attention, decision making process | - Social processes of encouragement, pressure and support - Planning, implementation - Adding equipment to the environment - Prompts, triggers and cues | - Schools will be supported via the external Support Officer to develop timetables and motivate teachers to supervise an organised PA at least 3 times per week. - Ideas and tips of how to implement this practice including case studies from other schools, will be placed on the PA4E1 program website. - Small equipment vouchers ($100AUD) will be provided to schools to support the delivery of organised activities. - Suggestions will be made for student council and sport captains to run recess and lunch activities, and existing teacher of playground duty to oversee (therefore no additional time demand on teachers). - Sample job descriptions for student leaders/sport captains will be placed on PA4E1 program website. Activities will be promoted to motivate students to attend. - At least one activity per week should specifically target girls. |
| Links with community PA providers | - Time available to make community links - Belief that students are already active enough outside of school - Belief that it is not the schools responsibility | - Motivation and goals - Memory, attention, decision making process - Knowledge - Social/Professional role and identity | - Contract - Prompts, triggers and cues - Information regarding behaviour, outcome - Social processes of encouragement, and support | - The in-School Champion and school PA4E1 committee will be responsible for developing links with community PA providers and promote new partnerships to students and families using three different mediums. - In-School Champions will be provided with data and information outlining the need and benefits of linking with the community (e.g. academic, social, mental health, fitness etc.) within the face to face training and resources placed on the PA4E1 program website. - The external Support Officer will prompt and remind the in-School Champion to develop community links. |
| Links with parents | - School messages are not read by parents/ limited parental engagement - Lack of knowledge related to:  1. PA guidelines 2. Strategies for supporting their child to be active 3. Benefits of role modelling PA by parents and siblings  - Lack of clarity who within the school is responsible for communication with parents | - Belief about consequences - Knowledge - Social/Professional role and identify - Memory, attention, decision making process | - Information regarding behaviour, outcome - Persuasive communication - Social processes of encouragement, pressure and support - Prompts, triggers and cues | - Example newsletter snippets will be developed by the project team and provided to schools via the PA4E1 program website. - The information to parents will use persuasive language on desired PA behaviour and positive outcomes. In addition to the newsletter snippets, a promotional video will be developed and placed on the program website and sent to program schools outlining what PA4E1 is, PA guidelines for adolescents, and motivating parents to role models. - In-School Champions and school admin staff will be charged with leading the communication of information to parents and provided with access to the program website to obtain resources, snippets and links to reliable PA information. - School Champion will receive prompts reminding them to regularly communicate PA information through a variety of mediums. |
